# Diagnostic Problems in C3 Glomerulopathy

**DOI:** 10.3390/biomedicines11041101

**Published:** 2023-04-05

**Authors:** Leszek Niepolski, Anna Czekała, Monika Seget-Dubaniewicz, Magdalena Frydrychowicz, Patrycja Talarska-Markiewicz, Angelika Kowalska, Jagoda Szmelter, Wiesława Salwa-Żurawska, Tomasz Sirek, Dawid Sobański, Beniamin Oskar Grabarek, Jakub Żurawski

**Affiliations:** 1 Department of Physiology, Poznan University of Medical Sciences, 60-567 Poznan, Poland; 2 Department of Clinical Pathology, Poznan University of Medical Sciences, 60-567 Poznan, Poland; 3 Department of Immunology, Poznan University of Medical Sciences, 60-567 Poznan, Poland; 4 Department of Immunobiology, Poznan University of Medical Sciences, 60-567 Poznan, Poland; 5 Department of Plastic Surgery, Faculty of Medicine, Academy of Silesia, 40-055 Katowice, Poland; 6 Department of Histology, Cytophysiology and Embryology, Faculty of Medicine, Academy of Silesia, 40-055 Katowice, Poland; 7 Department of Neurosurgery, Szpital sw. Rafala w Krakowie, 30-091 Krakow, Poland

**Keywords:** C3 glomerulopathy, kidney biopsy, electron microscopy, clinical manifestation, dense deposit disease (DDD), C3 glomerulonephritis (C3GN)

## Abstract

Background: C3 glomerulopathies (C3GN) are a group of rare kidney diseases associated with impaired complement regulation. The effects of this disease include the accumulation of complement C3 in the kidneys. Based on the clinical data, as well as light, fluorescence, and electron microscopy results, the diagnoses were verified. The study group consisted of biopsy specimens, which were obtained from 332 patients who were diagnosed with C3 glomerulopathy. In all cases, histopathological examinations were performed; deposits of complement C3 and C1q components, as well as the immunoglobulins IgA, IgG, and IgM, were identified using immunofluorescence. Furthermore, electron microscopy was also performed. Results: The histopathological examination results presented cases of C3GN (n = 111) and dense deposit disease (DDD; n = 17). The non-classified (NC) group was the most numerous (n = 204). The lack of classification was due to the poor severity of the lesions, even on the electron microscopic examination or in the presence of intense sclerotic lesions. Conclusions: In cases of suspected C3 glomerulopathies, we believe an electron microscopy examination is necessary. This examination is beneficial in mild-to-extremely-severe cases of this glomerulopathy, where the lesions are barely discernible when using immunofluorescence microscopy.

## 1. Introduction

Historically, membranoproliferative glomerulonephritis (MPGN) diagnosis has been mainly based on ultrastructural results, indicating three types of this disease [1]. Type I occurs with the presence of electron-dense subendothelial and mesangial deposits, together with the infiltration of mesangial cells and with monocytes penetrating and delaminating the basement membrane. In type II, the thickening of the basement membrane is described, with abundant deposits of very high electron densities. The presence of deposits is also observed in the mesangium and basement membrane of Bowman’s capsule and tubules. In type III, deposits are observed in both subendothelial and subepithelial regions, along with the doubling and rupture of the basement membrane [2,3].

The classification of MPGNs has changed in recent years. Due to a better understanding of the involvement of the C3 complement and a dysregulated alternative complement pathway, MPGN is presently known as C3 glomerulopathy (C3G). This change is associated with the predominant deposition of C3 within the mesangium or capillary wall, which is detected using immunofluorescence. Based on fluorescence and electron microscopy results, two types of C3G can be distinguished: dense deposit disease (DDD) and C3 glomerulonephritis (C3GN) [4,5,6,7,8,9]. In both forms of the disease, uncontrolled complement activity leads to progressive glomerulonephritis and scar formation with eventual chronic and irreversible damage that eventually leads to end-stage kidney failure [4,5,6].

Despite this new classification, C3 glomerulopathy is not a new disease [7]. DDD was previously known as MPGN type 2, while C3GN was historically classified as atypical type 1 and type 3 MPGN [8,9].

While MPGN was initially described using the classification proposed by Sethi [10], it was recently further reclassified into two diseases: immune complex MPGN (IC-MPGN) and C3 glomerulopathy (C3G). These classifications were based on immunofluorescence results in kidney biopsies, which were predominant or exclusive C3 deposits in C3G, complex immunoglobulins, and complement deposits in IC-MPGN. Based on the electron microscope results, C3G is further classified as DDD and C3GN. However, these updated classification criteria do not clarify how to differentiate the two diseases, which are clinically the best in terms of diagnosis, optimal treatment, and prognosis. In turn, the recent criteria for differentiating C3G from IC-MPGN are based on the predominance of C3 rather than immunoglobulins, which are assessed using fluorescence microscopy [11].

C3 glomerulopathies are a group of rare kidney diseases associated with impaired complement regulation, both in the fluid phase and in the glomerular microenvironment. The effects of this disease include the accumulation of complement C3 in the kidneys.

C3 glomerulopathy is a chronic progressive condition that mainly occurs in older children and young adults. The ages of the patients, according to the data, are usually between 10 and 30 years. Although rare, this type of inflammation can occur in the first few years of life and in the sixth decade and beyond. It represents 5–10% of all idiopathic nephrotic syndrome causes in children and adults [12,13].

In the United States, the incidence of C3 glomerulopathy may be as low as 5 cases per 1,000,000. In Europe, the incidence is estimated at 1 case per 1,000,000 [8].

The symptoms of C3 glomerulopathy range from asymptomatic hematuria and proteinuria to an acute course with classic symptoms of glomerulonephritis. Patients often have a history of hematuria and hypertension, which can be severe and may result from acute kidney injury or chronic kidney disease [10,14].

A kidney biopsy is necessary to establish the diagnosis. Immunofluorescence staining must show C3 deposits in the glomeruli that are at least two-fold larger than any other immunoreagent [15]. However, an electron microscopic examination makes it possible to distinguish between the two main subtypes of C3 glomerulopathy, which are DDD and C3GN [16].

In DDD, electron microscopy demonstrates the presence of osmophilic deposits within the basement membrane of glomerular capillaries, Bowman’s capsule, and tubules, as well as sometimes in the mesangial matrix [17]. In comparison, C3GN osmophilic deposits are mainly present in the mesangial area, often exhibiting an amorphic appearance [18].

The light microscopic images of DDD and C3GN are identical. The image’s most characteristic feature is the lobular structure of the glomeruli. Silver impregnation using the Jones stain reveals the presence of two or more silver-absorbent layers in the capillary wall. Between them, connecting “branchings” are sometimes present. Jones stains additionally reveal the segmental presence of “filaments” extending from the capillary basement membrane toward the epithelium [19,20].

## 2. Materials and Methods

### 2.1. Ethics

This retrospective, single-center study was conducted following the guidelines of the Helsinki Declaration and approved by the Bioethics Committee of the Medical University of Karol Marcinkowski in Poznan (KB 705/22). According to the statement of the Bioethics Committee, the study does not have the characteristics of a medical experiment and, by Polish law and good clinical practice (GCP), it is not subject to review by the Bioethics Committee. Written informed consent was obtained from all subjects involved in the study. In the case of minor patients, informed consent for their participation in the study was given by the parents or their legal guardians, with their consent corresponding to the minor’s implied consent. The minor was informed of the benefits and risks, and the consent of the parents or legal representative could be withdrawn at any time without harm to the minor. The investigator took into account the unambiguous wish of the minor (who were capable of expressing an opinion and evaluating information about the study) regarding their refusal to participate or their ability to withdraw from the study at any time. According to the provisions of Art. 25 of the Act on the Profession of Physicians and Dentists, the participation of a minor in a medical experiment is admissible only with the written consent of their statutory representative, i.e., both parents or a person appointed by the court.

Suppose a minor was over 13 years of age, by the provisions of Art. 25 of the Act on the Profession of Physician and Dentist and Art. 37h of the Pharmaceutical Law, in addition to the consent of the parents or legal representative. In that case, their written permission (the so-called double consent) was also required.

According to the European Medicines Agency’s (EMA’s) recommendation, oral consent was obtained from children over three years of age, and children aged 6–7 read and signed an information and informed consent form tailored to their understanding. Of course, if a child did not consent to participate or withdrew consent during the study, their opinion could not be disregarded.

In the case of minors under two years of age, written consent was given by the statutory representative, i.e., both parents and persons appointed by the court.

In a situation where the parents/statutory representatives did not consent to the minor’s participation in the study and the child gave such consent, the researcher, to the best of their knowledge, made a judgment about the best therapeutic option for the patient, and the guardianship court resolved the case.

### 2.2. Characteristics of the Study Group

The study group consisted of biopsy material obtained from 332 (246 adults and 86 children) patients in whom a diagnosis of C3 glomerulopathy was initially suspected on the basis of clinical symptoms, such as hypertension, and/or proteinuria, and/or nephrotic syndrome, and/or hematuria. In these cases, the biopsy results qualified for further treatment. The fresh kidney biopsy was divided into three parts in order to perform simultaneous diagnostic studies under light, fluorescence, and electron microscopy.

### 2.3. Examination of the Biopsy Material

#### 2.3.1. Histopathological Examination

Paraffin slides were stained with a hematoxylin and eosin kit (Abcam, Cambridge, MA, USA), which included a bluing reagent, eosin Y solution, and hematoxylin (modified Lillie’s Mayer’s solution), and then impregnated using a Jones stain kit (Abcam, Cambridge, MA, USA).

#### 2.3.2. Immunofluorescence Studies

The tissue material collected from the kidney biopsy was rapidly frozen in liquid nitrogen and cut to a thickness of 5 µm using a cryostat (Thermo Scientific, Cheshire, UK). Biopsy slides were fixed in a cold 1:1 mixture of alcohol and acetone at 4 °C for 5 min. The slides were then dried at room temperature for 5 min and washed three times using phosphate-buffered saline (PBS, pH 7.4; Thermo Fisher Scientific, Waltham, MA, USA). The samples were incubated with antibodies against IgA, IgG, and IgM, as well as C3 and C1q, which are complement proteins. They were then labeled with fluorescein 5-isothiocyanate (FITC; Thermo Fisher Scientific, Waltham, MA, USA) in the dark at room temperature. A 1:20 dilution of antibodies was used. Following incubation, the slides were washed three times in PBS and embedded in a glycerol medium. The presence, composition, and location of immunoglobulins and complement protein deposits were assessed using an Olympus BX43 research microscope with an EPI fluorescence kit (Olympus, Tokyo, Japan).

#### 2.3.3. Electron Microscopic Studies

The samples for ultrastructural studies were fixed in a 3.6% buffered glutaraldehyde solution at pH 7.4 (Poly Scientific R&D Corporation, Bay Shore, NY1176, USA). They were then embedded in an Epon 812 epoxy resin (Sigma Aldrich, Poznan, Poland) and sliced into 1 µm-thick slices using an ultramicrotome. Subsequently, they were stained with toluidine blue and examined using a light microscope. Such ultra-thin sections were examined using electron microscopy. The photographic documentation was collected using an Opton 900 transmission electron microscope (Zeiss, Oberkochen, Germany).

**Figure 1:**
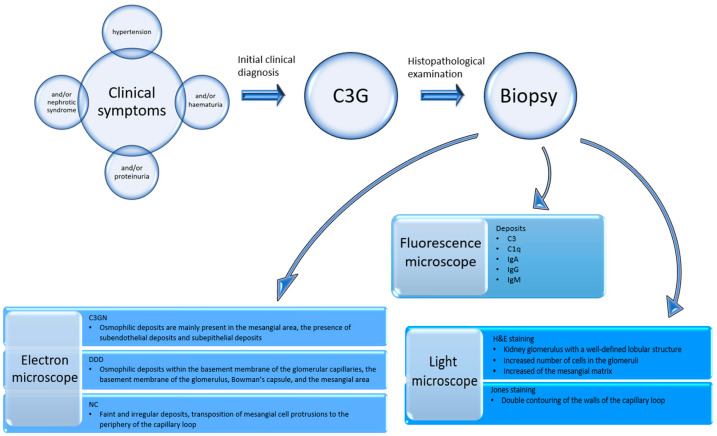
Diagram of the study flow. G3GN, C3 glomerulonephritis; DDD, dense deposit disease; NC, non-classified group; C3, C1q, complement component; IgA, immunoglobulin A; IgG, immunoglobulin g; IgM, immunoglobulin M.

The diagram of the above study flow description is shown in Figure 1.

### 2.4. Clinical Definitions

Hematuria is the presence of red blood cells in the urine in numbers >3 in the field of view of the urine sediment. Proteinuria is the presence of 0.5–3.5 g/day of protein in the urine. Nephrotic syndrome occurs when urinary protein excretion exceeds 3.5 g/24 h or 50 mg/kg/24 h, accompanied by peripheral edema and hypoalbuminemia of ˂3.5 g/dL

Acute kidney injury (AKI) is a set of symptoms associated with the sudden deterioration of kidney function. This occurs as a result of kidney waste being insufficiently excreted from the body’s metabolic products. Furthermore, in AKI, water and electrolyte balance deteriorates, and acute uremia develops. AKI is manifested by oliguria or anuria, and azotemia (evidenced in the increases in the concentration of non-protein nitrogen and creatinine in the blood, which is usually due to a decrease in glomerular filtration).

Hypertension can range from grade I hypertension (systolic 140–159 and/or diastolic 90–99), grade II hypertension (systolic 160–179 and/or diastolic 100–109), grade III hypertension (systolic ≥180 and/or diastolic ≥110), and isolated systolic hypertension (≥140 systolic and <90 diastolic).

Progression is an exacerbation of the disease in which the patient’s condition continues to deteriorate. This includes symptoms that have worsened. In addition, these also include hematuria, proteinuria, hypertension or nephrotic syndrome, steroid resistance, and steroid dependence. The progression of histopathological changes is associated with the presence of histological markers indicating the development of the disease. These include glomerulosclerosis, the presence of deposits in the mesangial area, and the glomerular basement membrane, among others.

### 2.5. Statistical Analysis

Statistical analysis was performed in R software, version 4.0.5. The relationship between inflammation type and sex, clinical symptoms, rebiopsies, and progression was evaluated using Fisher’s exact test. A significance level of α = 0.05 was used (*p* < 0.05).

## 3. Results

### 3.1. Study Group

**Figure 2:**
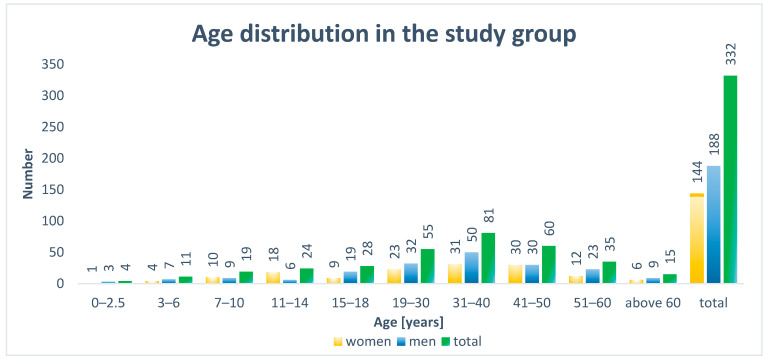
Age distribution in the study group.

The examined group of 246 adults included 102 females and 144 males. In turn, the 86 examined children comprised 42 females and 44 males. Among the adults, patients between 31 and 40 years of age were the most numerous age group (n = 81). In the smaller group of children, no predominant age group was noted (Figure 2).

### 3.2. Patient Group Classification

C3GN was determined in 111 patients. In contrast, DDD was diagnosed in 17 patients. The unclassified group (NC) was the most numerous with 204 patients.

In children, a statistically significant relationship between gender and the type of inflammation was confirmed (*p* = 0.008). There were more males in the C3GN group (71%) than in the DDD group (38%) or the unspecified type of group (37%).

In contrast, in the adult group, no significant relationship between inflammation type and gender was confirmed (*p* = 0.414) (Table 1). The reasons for the lack of classification included the faintness of lesions on fluorescence microscopy, as well as in regard to electron microscopic examination or the presence of intense sclerosis.

Furthermore, the deposits were sometimes small and variably distributed. However, in all these cases, the transposition of mesangial cell protuberances to the periphery of the loop was observed. Similarly, in cases with significant progression (sclerosis), transposition was observed in at least several capillary loops.

### 3.3. Clinical Symptoms

In the group of adult patients, the most frequent clinical symptoms, regardless of the type, were proteinuria (n = 147) and nephrotic syndrome (n = 62). In a significant number of patients, they were accompanied by other symptoms, such as hematuria and hypertension. In women, a statistically significant relationship between the type of inflammation and the presence of proteinuria with hematuria was confirmed (*p* = 0.028). This symptom was present in 67% of women with DDD (2/3 individuals) compared with 5.6% of women with C3GN (2/36 individuals) and 11% of women with an unspecified disease type (7/63 individuals). In men, a statistically significant association between the type of inflammation and the presence of proteinuria was confirmed (*p* = 0.008). Proteinuria affected 50% of men with DDD (3/6 subjects) compared with 20% of men with C3GN (8/40 subjects) and 8.2% of men with an unspecified disease type (8/98) (Table 2).

In turn, in the group of children, the most frequent clinical symptoms, regardless of the type, were nephrotic syndrome (n = 39) and proteinuria (n = 37).

In females, the inflammatory type was significantly associated with the occurrence of nephrotic syndrome and hypertension (*p* = 0.009). This symptom affected 10% of girls with C3GN (1/10 subjects), no girls with an unspecified disease type (0/27 subjects), and 40% of girls with DDD (2/5 subjects).

**Figure 3:**
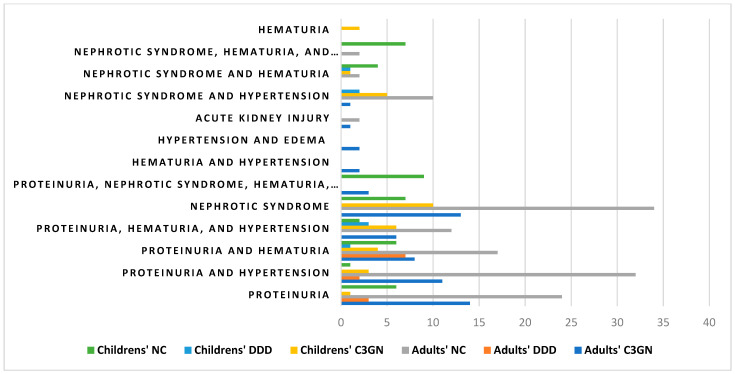
Number of cases in each group diagnosed with a particular symptom or symptoms.

In males, on the other hand, a statistically significant relationship was confirmed between the type of inflammation and the occurrence of proteinuria with hematuria and hypertension (*p* = 0.003). A statistically significant relationship was also observed between nephrotic syndrome with hematuria and hypertension (*p* = 0.047). Proteinuria with hematuria and hypertension was present in all males with DDD (3 subjects) vs. 12% of males with C3GN (3/25 subjects) and 5.9% of males with an unspecified disease type (1/17 subjects). In contrast, nephrotic syndrome with hematuria and hypertension affected 24% of boys with the indeterminate type of inflammation (4/17 subjects) vs. none with C3GN (0/25 subjects) or DDD (0/3 subjects). A list of clinical symptoms in both adults and children is presented in Table 2 and Figure 3. Additionally, a detailed list of the clinical symptoms in both adults and children is presented in Table A1 and Table A2.

### 3.4. Histopathological Examination

**Figure 4:**
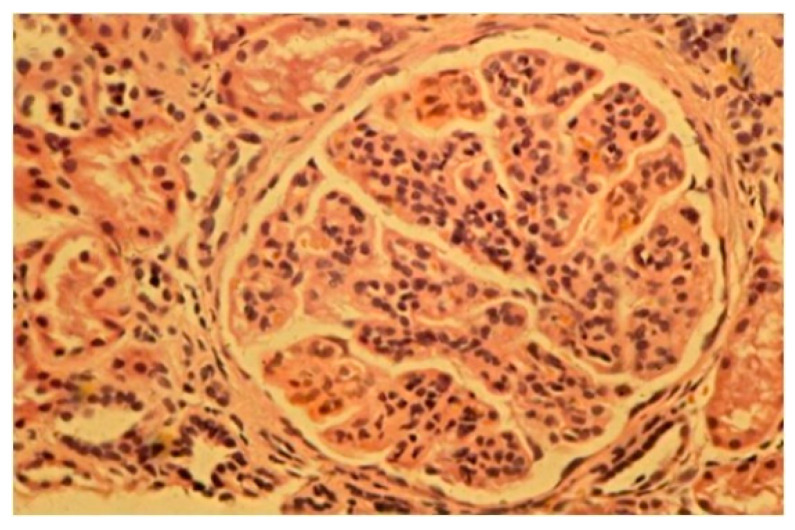
Kidney glomerulus with an increased number of cells and a well-defined lobular structure. H&E, 100× magnification.

**Figure 5:**
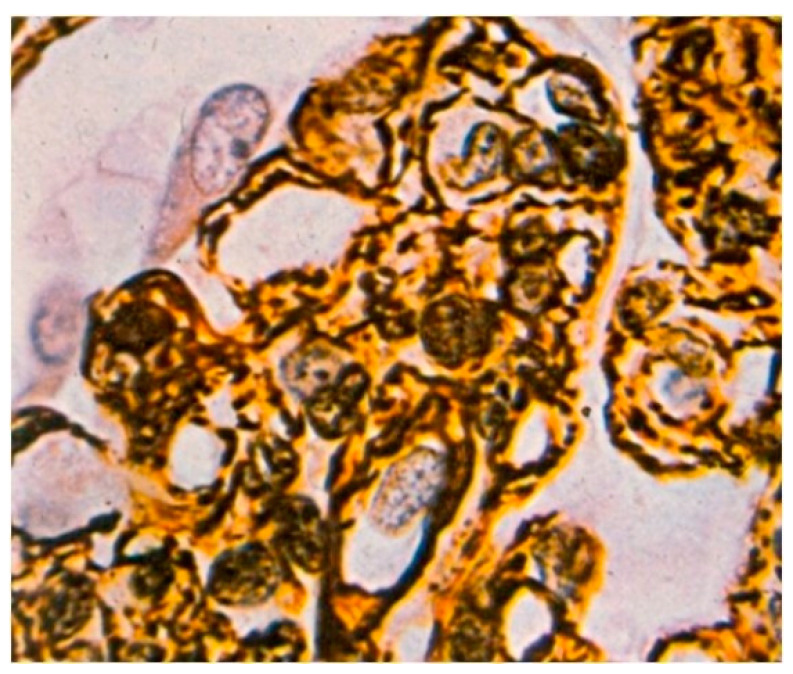
Double-contouring of the walls of the capillary loops. Impregnation with silver-containing salt according to a Jones stain, 400× magnification.

Light microscopy showed an increased number of cells in the glomeruli and an adventitia of the mesangial matrix. The kidney glomeruli were characterized by a lobular structure (Figure 4). Jones’ stain method highlighted the double-contouring of the capillary loop walls (Figure 5).

### 3.5. Immunofluorescence Studies

In the immunofluorescence study, C3 (n = 211), IgM (n = 204), and C1q (n = 158) were most frequently observed in adult patients.

In turn, IgA (n = 50) and IgG (n = 50) were observed in children, while IgM (n = 37) was detected slightly less frequently. C3 was observed in 81 child patients, while C1q was noted in 44.

In C3GN, some cases only presented C3 deposits. In some patients, deposits of IgA, IgG, IgM, and C1q were also observed, but with much lower fluorescence intensities compared with C3.

In DDD, C3 deposits were present in the basement membranes of the glomeruli.

### 3.6. Electron Microscopic Examination

#### 3.6.1. C3GN

**Figure 6:**
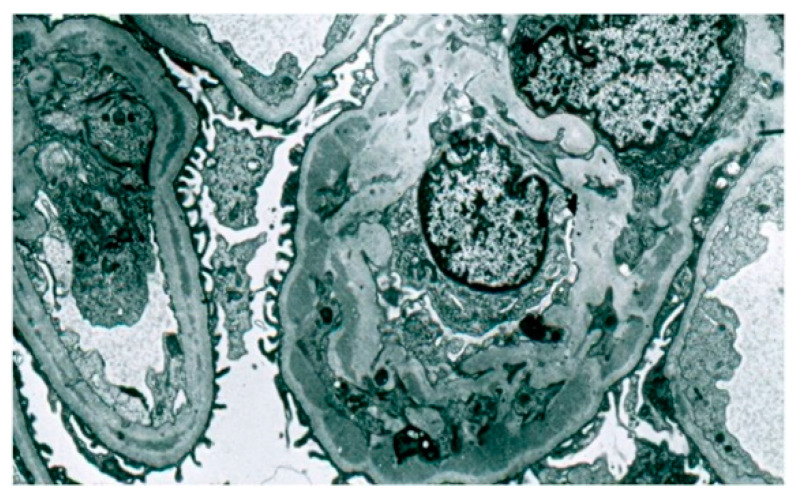
A case of C3GN. Electron microscopic image of the glomerulus, with transposition of mesangial cell protuberances to the periphery of capillary loops and with a ‘double’ basement membrane. Subendothelial deposits. Electron microscopy, 8750× magnification.

**Figure 7:**
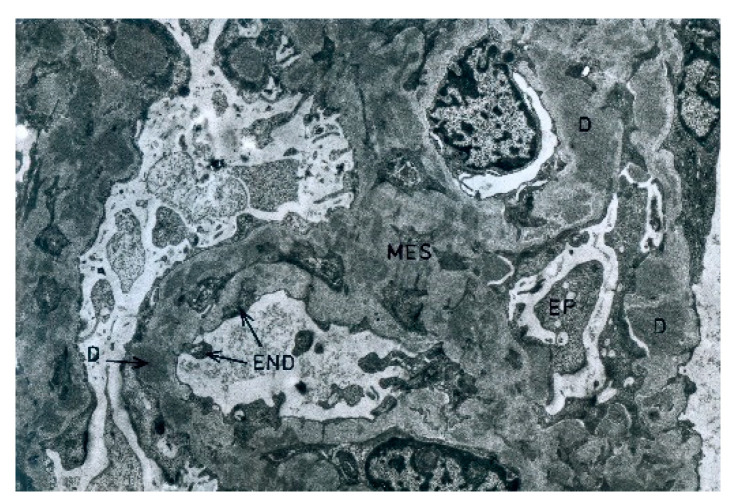
The case of C3GN. Electron microscopic image of the glomerulus with transposition of mesangial cell protrusions to the periphery of the capillary loop and presence of subepithelial deposits. Electron microscopy, 8750× magnification. Mes, mesangium; EP, epithelium; END, endothelium; D, deposits.

During the electron microscopic examination, the transposition of mesangial cell protuberances to the periphery of the capillary loops, as well as the presence of a ‘double’ basement membrane resulting from the presence of subendothelial deposits, were visible in the glomeruli (Figure 6). Glomeruli, with transposition of mesangial cell protuberances to the periphery of the loop, as well as in the presence of subepithelial deposits, were also noted (Figure 7).

#### 3.6.2. DDD

**Figure 8:**
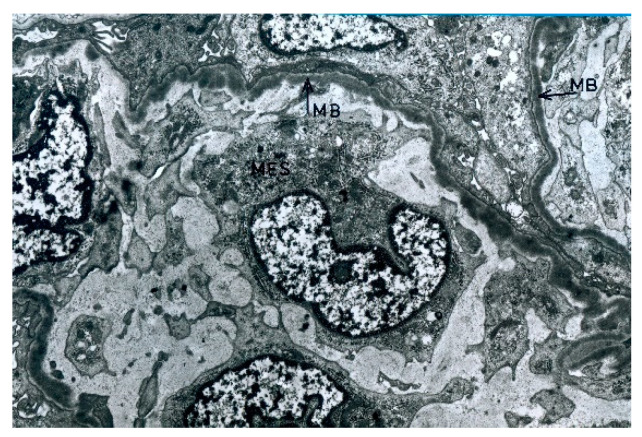
A case of DDD. Electron microscopic image of a glomerulus with a markedly thickened basement membrane structure, as well as a transposition of mesangial cell protrusions. Electron microscopy, 7500× magnification. MB, basement membrane; Mes, mesangium.

The electron microscopic examination revealed osmophilic deposits within the basement membrane of the glomerular capillaries (Figure 8). Deposits were also observed in the basement membrane of the glomerulus, Bowman’s capsule, and mesangial area.

#### 3.6.3. Non-Classification Group

The non-classification criteria in the electron microscopy examinations included low-severity cases of lesions or the presence of intense sclerosis.

The electron and fluorescence microscopy showed faint and irregular deposits in these cases. However, in all of these cases, the transposition of mesangial cell processes to the periphery of the loop was found. Similarly, in advanced cases (sclerosis), transposition was found in at least a few of the capillary loops.

This group included several patients whose tissue material was not secured for electron microscopy. However, through the use of a light microscope, a lobular structure of the glomeruli was observed, and a double outline of the basement membrane was found in at least some of the capillary loops. These results were obtained in the preparations that were impregnated with silver-containing salt, as per a Jones staining. In addition, when viewing the material through a fluorescence microscope, the presence of deposits was small.

### 3.7. Rebiopsies and Progression

All kidney biopsies were clinically indicated. In adults, this was conducted due to diagnosed nephrotic syndrome, proteinuria, or isolated hematuria, and in children, this was due to steroid-resistant nephrotic syndrome. All rebiopsies were performed for clinical indications, which were due to a lack of remission.

Rebiopsies were only performed on 31 adult patients (31–49 years) and 30 children (5–11 years). Progression was observed in 15 adult patients and 17 children. In cases where there was no indication of lesion severity, rebiopsies were performed early after the first biopsy in order to establish a definitive diagnosis. This was performed if the first biopsy yielded unrepresentative material, or if the lesions were of low severity.

#### 3.7.1. Rebiopsies and Progression: Women

In a group of 15 women who underwent rebiopsies, 5 were diagnosed with C3GN and 10 with unclassified lesions. Both patients with C3GN showed progression 2 years after the first biopsy, with one patient showing a small increase in lesion severity when compared with the first examination. Progression markers were not observed in the other three patients with C3GN, one of whom had a relatively late rebiopsy, which was 7 years after the first examination.

In the group of 10 patients with unclassified lesions, there were four with no signs of progression (one of whom had a rebiopsy 6 years after, and the other 7 years after the first biopsy). Progression was observed in the six other patients: in two after 1 year, in another two after 2 years, and in one after 7 years.

#### 3.7.2. Rebiopsies and Progression: Males

In the male group, rebiopsies were performed on 16 patients, including 2 with C3GN and 14 with lesions of an unclassified type. In one patient with C3GN, a rebiopsy was performed very early after the first examination in which a definitive diagnosis could not be established; in the other, progression was identified after 3 years. The group with unclassified lesions included eight patients with no evidence of progression (seven at 2 years, and one at 4 years after the first examination). In the group of seven patients with evidence of progression, it was observed in three after 1 year, in another two after 3 years, in one after 5, and in one after 27 years from the first biopsy. No statistically significant relationship was confirmed between the type of inflammation, the frequency of rebiopsies, or the presence of progression in adult men and women (Table 3).

#### 3.7.3. Rebiopsies and Progression: Female Children

In the female child group, progression was observed in eight patients with C3GN (n = 3) and unclassified lesions (n = 5). Progression was diagnosed after 3 years in two patients with C3GN, and after 2 years in one 7-year-old patient. In turn, among the patients with an unclassified lesion type, progression was diagnosed after 2 years in two, after 4 years in one, after 5 years in one, and in one patient after a few months from the first biopsy. This last rebiopsy was performed because of rapidly progressing kidney failure.

It is notable that in the group of girls with C3GN, there was a seven-year-old girl who underwent a rebiopsy after one year without finding progression and died shortly afterward due to kidney failure.

For girls, there was a statistically significant association between the inflammation type and the rate of rebiopsies (*p* = 0.036), as well as the rate of progression/no progression (*p* = 0.036). Rebiopsy was performed in 60% of girls with C3GN (6/10 patients) when compared with 40% of patients with DDD (2/5 patients) and 19% of patients with the unspecified inflammatory type (5/27 patients). A biopsy with progression occurred in all girls with the indeterminate inflammatory type (five patients), in 50% of patients with C3GN (3/6 patients), and in no patients with DDD (0/2 patients). Detailed data are presented in Table 4.

#### 3.7.4. Rebiopsies and Progression: Male Children

In the male child group, progression was observed in nine patients with C3GN (n = 5) and those with unclassified lesions (n = 4).

In patients with C3GN lesions, progression was observed after 1 year in one patient and after 2 years in three patients. One patient showed progression 3 years after the first biopsy. In the unclassified type of group, progression was observed in two patients at 2 years, in one at 3 years, and in one at 4 years after the first biopsy.

In male children, no statistically significant relationship was confirmed between the type of inflammation, rates of rebiopsies, and progression/no progression. Detailed data are presented in Table 4.

## 4. Discussion

In the cases of C3 glomerulopathy, there are several issues that have not yet been fully clarified in the literature. The classification of MPGN has changed in recent years. As a consequence of a better understanding of the pathomechanisms of this inflammation and the development of fluorescence microscopy studies, it was renamed C3 glomerulopathy (C3G). Therefore, in the cases presented in our study, we also paid attention to the electron microscopic changes [4,8,21].

The literature most commonly mentions nephrotic and nephritic syndromes as the typical clinical presentation of C3GN. For DDD, nephrotic syndrome and subrenal proteinuria are primarily mentioned [22]. In our material, regardless of type, proteinuria (n = 147) and nephrotic syndrome (n = 62) predominated by far. Proteinuria and nephrotic syndrome were often accompanied by hematuria and/or hypertension. In the group of children studied, nephrotic syndrome was present in 39 patients. In contrast, proteinuria was present in 37 adults.

It is noteworthy that the group of girls included a seven-year-old patient with C3GN lesions, in whom a rebiopsy was performed one year after the first biopsy and yielded no indications of progression. Nonetheless, the child died soon after from kidney failure. This may have been related to the process known to occur in patients with C3GN, whereby the condition results in the development of end-stage kidney failure in a relatively short period. In this situation, such patients require dialysis or a kidney transplant [23]. Patients with C3 glomerulopathy usually present proteinuria and hematuria. Although all age groups are affected, their disease triggers vary. In children and young adults, C3 glomerulopathy is often preceded by an upper respiratory tract infection [24]. Furthermore, the mean age at diagnosis is lower in patients with DDD than in those diagnosed with C3GN. DDD is also less common, with it being diagnosed approximately half as often as C3GN [25].

In our data, the highest number of adult patients were between the ages of 30 and 50 years, followed by those aged 19 to 30 years. The children group was more diverse regarding their age. The youngest child was 2 years old at the time of the diagnosis, while the largest group was aged 14–17 years.

An electron microscopic examination permits the observation of the displacement of mesangial cell filaments toward the periphery of capillary loops and to the characteristic distribution of deposits. The displaced filaments are located between the basement membrane and the endothelial cells. They are often separated from the endothelium by a layer resembling the structure of the basement membrane. The presence of this layer results in a double-contoured image, which is observed in the histological slides that are impregnated with silver salts. In C3GN, electron-dense deposits are often observed in this layer. However, they are not always accompanied by the displacement of the filaments. Sometimes, the deposits are present in one loop and displaced filaments in another. The deposits are usually small-to-medium-sized and are rarely large. Within the mesangium, deposits are usually present in the early stages of the disease. Moreover, sparse subepithelial deposits are present in 30–50% of cases [26].

In contrast, an important ultrastructural feature of DDD is the presence of osmophilic deposits within the basement membrane of glomerular capillaries [13,27].

The final indication for a kidney biopsy is usually the presence of persistent hematuria and/or proteinuria with low serum C3 levels [28,29,30].

The most important adverse outcome associated with a diagnosis of C3 glomerulopathy is its progression to end-stage kidney disease (ESKD), which occurs within 10 years of diagnosis in approximately 70% of children and 30–50% of adults [31].

In the immunomorphological examination of the adult group, similar to those cited in the literature, the most frequent results include the presence of C3 (n = 211), IgM (n = 204), and C1q (n = 158). However, IgA and IgG expressions were not reported in this group. In contrast, in the studied group of children, C3 (n = 81), IgA and IgG (n = 50), C1q (n = 44), and IgM (n = 37) were most frequently observed. The results of C3 and C1q assays are similar in both groups.

Based on the results, it can be speculated that the patients we initially included in the NC group could be diagnosed with IC-MPGN due to the presence of IgM in all patients in the adult patient group. In contrast, in children, we observed IgA and IgG in 50 patients and IgM in 37 patients.

Most literature sources state that their fluorescence microscopy results were obtained from frozen material. Meanwhile, there are some reports of high rates of suspected false-negative monoclonal immunoglobulin staining in routine immunofluorescence assays. In such cases, paraffin immunofluorescence studies should be performed to avoid misdiagnoses. Furthermore, it may be worthwhile to perform these studies in our group of unclassified patients for definitive verification [32].

In contrast, some authors indicate the opposite occurrence. The final assessment of C3 staining intensity may also be influenced by the immuno-staining technique used and the tissue preparation methods employed. Immunofluorescence in frozen sections, as well as in formalin-fixed and paraffin-embedded tissues, can exhibit varying sensitivity rates. Hence, it was reported that the immunofluorescence in the frozen sections showed greater sensitivity than in formalin-fixed and paraffin-embedded tissues [33]. 

The presence of IgG and C3 is frequently observed in patients with post-infectious glomerulonephritis (PIGN). It is very difficult to differentiate these two disease entities on the basis of histological features alone. The clinical course and laboratory results ultimately distinguish these diseases. Most patients with PIGN recover normal kidney functions. Further, hematuria and proteinuria disappear within a few weeks without therapeutic intervention [34].

It is difficult for us to address the issue of rebiopsies due to their relatively small number in our sample of 31 adult patients and 30 children. However, in the group of adult patients, based on the rebiopsy results obtained, histological progression could be detected in 3 patients with C3GN and 12 with NC. In the group of affected children, histological progression was observed in 17:8 (male:female) with C3GN and 9 with NC.

Despite the numerous reports on both the diagnosis and treatment of C3G, the optimal treatment for this disease has still not been established. This results from the fact that continuous and uncontrolled complement activity, which is characteristic of C3G, induces glomerulonephritis and leads to subsequent fibrosis. This, in turn, causes chronic and irreversible kidney damage.

The greatest diagnostic difficulties appeared in the non-classified group (NC). These difficulties resulted primarily from the uncharacteristic picture of changes observed, both in the electron microscope and in the fluorescence microscope; further, we found a few irregular deposits. On the other hand, in these cases, we observed a transposition of mesangial cell protrusions onto the periphery of the loop. This was an electron microscope image that would suggest C3GN. We saw the value of electron-microscopic examination in these cases; at the same time, we could not determine the distribution of C3 complement deposits in the glomeruli through the use of a fluorescence microscope.

### Limitations of the Study

Of course, the study we conducted had limitations. First, the study was retrospective and the obtained data were from just one center. Second, it would be interesting to supplement the data we obtained with the demographic and anthropometric data of the study participants. Third, the study was limited by the relatively small number of participants in each subgroup. Therefore, we were unable to run a multivariate logistic regression, which would have yielded more conclusions. In the case of children, we only had 42 females and 44 males, with less than 30 in any inflammation-type subgroup, which was not enough for a multivariate model. For the adults, we had very small DDD subgroups (three females and six males); once again, the numbers were too low for any modeling. 

A fourth limitation of the study was the tissue assessment methods used. The result of immunofluorescence staining was affected by the preparation of the material for analysis [3,32,33]. Furthermore, it was very difficult to differentiate between C3GN and DDD based solely on the electron microscopy results. Fifth, the updated classification criteria do not clarify how to differentiate between the two diseases, which are clinically the best in terms of diagnosis, optimal treatment, and prognosis.

## 5. Conclusions

Based on the literature data and our observations, we believe that given the rarity of C3 glomerulopathy, it is necessary to publish the results of even single cases of this glomerulopathy.

In cases of suspected C3G, we believe that an electron microscopic examination is necessary. This examination is helpful in cases with sparse or extremely severe lesions of this glomerulopathy, as they are hardly discernible when using immunofluorescence microscopy. 

It is very difficult to differentiate between C3GN and DDD based on electron microscopy results only. However, in our opinion, in these cases, it is possible to make a preliminary diagnosis of C3GN. For a full diagnosis, however, both clinical data and the results of the electron, fluorescence, and light microscopy are needed. Unfortunately, we did not find similar observations in the available literature. Therefore, prospective multicenter studies involving nephrologists, pathologists, and clinical geneticists may prove useful.

We conclude that it is necessary to perform an electron microscopic examination to determine the type of inflammation (C3GN, DDD, and IC-MPGN), as well as confirm the clinical data.

## Figures and Tables

**Table 1:** Relationship between the type of inflammation and gender.

	Total	Inflammation Type			*p*-Value
		C3GN	DDD	NC	
Children					
Females	42	10 (28.6%)	5 (62.5%)	27 (62.8%)	0.008
Males	44	25 (71.4%)	3 (37.5%)	16 (37.2%)	
Adults					
Females	102	36 (47.4%)	3 (33.3%)	63 (39.1%)	0.414
Males	144	40 (52.6%)	6 (66.7%)	98 (60.9%)	

The data are presented as the number of individuals n (% of total group/type of inflammation). Inflammation types were compared using Fisher’s exact test. Abbreviations: DDD, dense deposit disease; C3GN, C3 glomerulonephritis; NC, non-classification criteria.

**Table 2:** Clinical symptoms in adults and children.

Group of Patients	Gender	Total	Inflammation Type			*p*-Value
			C3GN	DDD	NC	
Adults	Females, n	102	36 (35.30%)	3 (2.04%)	63 (61.76%)	0.310
	Males, n	144	40 (27.78%)	6 (4.16%)	98 (68.06%)	
Children	Females, n	42	10 (23.81%)	5 (11.90%)	27 (64.29%)	0.011
	Males, n	44	25 (56.82%)	3 (4.44%)	17 (38.64%)	

The data are presented as the number of individuals n (% of total group/type of inflammation). Inflammation types were compared using a chi-square test. Abbreviations: DDD, dense deposit disease; C3GN, C3 glomerulonephritis; NC, non-classification group.

**Table 3:** Relationship between the type of inflammation, rebiopsy, and progression in adults.

	Total	Inflammation Type			*p*-Value
		C3GN	DDD	NC	
Females, n	102	35	4	63	
Rebiopsy	15 (14.7%)	5 (14.3%)	0 (0.0%)	10 (15.9%)	>0.999
No rebiopsy	87 (85.3%)	30 (85.7%)	4 (100.0%)	53 (84.1%)	
Biopsy: *					
Progression	8 (53.3%)	2 (40.0%)	0 (0.0%)	6 (60.0%)	0.608
No progression	7 (46.7%)	3 (60.0%)	0 (0.0%)	4 (40.0%)	
Males, n	144	40	6	98	
Rebiopsy	16 (11.1%)	2 (5.0%)	0 (0.0%)	14 (14.3%)	0.295
No rebiopsy	128 (88.9%)	38 (95.0%)	6 (100.0%)	84 (85.7%)	
Biopsy: *					
Progression	7 (43.8%)	1 (50.0%)	0 (0.0%)	6 (42.9%)	>0.999
No progression	9 (56.3%)	1 (50.0%)	0 (0.0%)	8 (57.1%)	

The data are presented as the number of individuals n (% of total group/type of inflammation). *: % calculated in relation to the number of rebiopsies. Inflammation types were compared using Fisher’s exact test. The calculated *p*-value > 0.999 was from Fisher’s exact test. This test was possible in the absence of observations and the obtained *p*-value could be interpreted as in any other case. Therefore, there was no statistically significant difference in the incidences. DDD, dense deposit disease; C3GN C3 glomerulonephritis; NC, non-classification criteria.

**Table 4:** Relationship between the type of inflammation, rebiopsy, and progression in children.

	Total	Inflammation Type			*p*-Value
		C3GN	DDD	NC	
Females, n	42	10	5	27	
Rebiopsy	13 (31.0%)	6 (60.0%)	2 (40.0%)	5 (18.5%)	0.036
No rebiopsy	29 (69.0%)	4 (40.0%)	3 (60.0%)	22 (81.5%)	
Biopsy: *					
Progression	8 (61.5%)	3 (50.0%)	0 (0.0%)	5 (100.0%)	0.036
No progression	5 (38.5%)	3 (50.0%)	2 (100.0%)	0 (0.0%)	
Males, n	44	9	3	16	
Rebiopsy	17 (38.6%)	12 (48.0%)	0 (0.0%)	5 (31.3%)	0.228
No rebiopsy	27 (61.4%)	13 (52.0%)	3 (100.0%)	11 (68.8%)	
Biopsy: *					
Progression	9 (52.9%)	5 (41.7%)	0 (0.0%)	4 (80.0%)	0.294
No progression	8 (47.1%)	7 (58.3%)	0 (0.0%)	1 (20.0%)	

The data are presented as the number of individuals n (% of total group/type of inflammation). *: % calculated in relation to the number of rebiopsies. Inflammation types were compared using Fisher’s exact test. DDD, dense deposit disease; C3GN C3 glomerulonephritis; NC, non-classification criteria.

## Data Availability

The data used to support the findings of this study are included within the article.

## Appendix A

**Table A1:** A detailed list of the clinical symptoms in adults.

	Total	Inflammation Type			*p*-Value
		C3GN	DDD	NC	
Females, n	102	36	3	63	
Proteinuria	24 (23.5%)	6 (16.7%)	1 (33.3%)	17 (27.0%)	0.374
Proteinuria and hypertension	19 (18.6%)	11 (30.6%)	0 (0.0%)	8 (12.7%)	0.069
Proteinuria and hematuria	11 (10.8%)	2 (5.6%)	2 (66.7%)	7 (11.1%)	0.028
Proteinuria, hematuria, and hypertension	6 (5.9%)	0 (0.0%)	0 (0.0%)	6 (9.5%)	0.182
Nephrotic syndrome	24 (23.5%)	9 (25.0%)	0 (0.0%)	15 (23.8%)	>0.999
Proteinuria, nephrotic syndrome, hematuria, and hypertension	3 (2.9%)	3 (8.3%)	0 (0.0%)	0 (0.0%)	0.128
Hematuria and hypertension	0 (0.0%)	0 (0.0%)	0 (0.0%)	0 (0.0%)	>0.999
Hypertension and edema	1 (1.0%)	1 (2.8%)	0 (0.0%)	0 (0.0%)	0.382
Acute kidney injury	3 (2.9%)	1 (2.8%)	0 (0.0%)	2 (3.2%)	>0.999
Nephrotic syndrome and hypertension	5 (4.9%)	1 (2.8%)	0 (0.0%)	4 (6.3%)	0.699
Nephrotic syndrome and hematuria	1 (1.0%)	0 (0.0%)	0 (0.0%)	1 (1.6%)	>0.999
Nephrotic syndrome, hematuria, and hypertension	1 (1.0%)	0 (0.0%)	0 (0.0%)	1 (1.6%)	>0.999
Males, n	144	40	6	98	
Proteinuria	19 (13.2%)	8 (20.0%)	3 (50.0%)	8 (8.2%)	0.008
Proteinuria and hypertension	39 (27.1%)	13 (32.5%)	2 (33.3%)	24 (24.5%)	0.569
Proteinuria and hematuria	17 (11.8%)	6 (15.0%)	1 (16.7%)	10 (10.2%)	0.548
Proteinuria, hematuria, and hypertension	12 (8.3%)	6 (15.0%)	0 (0.0%)	6 (6.1%)	0.200
Nephrotic syndrome	23 (16.0%)	4 (10.0%)	0 (0.0%)	19 (19.4%)	0.260
Proteinuria, nephrotic syndrome, hematuria, and hypertension	0 (0.0%)	0 (0.0%)	0 (0.0%)	0 (0.0%)	>0.999
Hematuria and hypertension	2 (1.4%)	2 (5.0%)	0 (0.0%)	0 (0.0%)	0.158
Hypertension and edema	1 (0.7%)	1 (2.5%)	0 (0.0%)	0 (0.0%)	0.319
Acute kidney injury	0 (0.0%)	0 (0.0%)	0 (0.0%)	0 (0.0%)	>0.999
Nephrotic syndrome and hypertension	6 (4.2%)	0 (0.0%)	0 (0.0%)	6 (6.1%)	0.269
Nephrotic syndrome and hematuria	1 (0.7%)	0 (0.0%)	0 (0.0%)	1 (1.0%)	>0.999
Nephrotic syndrome, hematuria, and hypertension	1 (0.7%)	0 (0.0%)	0 (0.0%)	1 (1.0%)	>0.999

The data are presented as the number of individuals n (% of total group/type of inflammation). Inflammation types were compared using Fisher’s exact test. The calculated *p*-value > 0.999 was from Fisher’s exact test. This test was possible in the absence of observations and the obtained *p*-value could be interpreted as in any other case. Therefore, there was no statistically significant difference in the incidence. Abbreviations: DDD, dense deposit disease; C3GN C3 glomerulonephritis; NC, non-classification criteria.

**Table A2:** A detailed list of the clinical symptoms in adults.

	Total	Inflammation Type			*p*-Value
		C3GN	DDD	NC	
Females, n	42	10	5	27	
Proteinuria	6 (14.3%)	1 (10.0%)	0 (0.0%)	5 (18.5%)	0.833
Proteinuria and hypertension	2 (4.8%)	2 (20.0%)	0 (0.0%)	0 (0.0%)	0.064
Proteinuria and hematuria	6 (14.3%)	0 (0.0%)	1 (20.0%)	5 (18.5%)	0.403
Proteinuria, hematuria, and hypertension	4 (9.5%)	3 (30.0%)	0 (0.0%)	1 (3.7%)	0.068
Nephrotic syndrome	7 (16.7%)	1 (10.0%)	1 (20.0%)	5 (18.5%)	>0.999
Proteinuria, nephrotic syndrome, hematuria, and hypertension	6 (14.3%)	0 (0.0%)	0 (0.0%)	6 (22.2%)	0.259
Nephrotic syndrome and hypertension	3 (7.1%)	1 (10.0%)	2 (40.0%)	0 (0.0%)	0.009
Nephrotic syndrome and hematuria	5 (11.9%)	1 (10.0%)	1 (20.0%)	3 (11.1%)	0.794
Nephrotic syndrome, hematuria, and hypertension	3 (7.1%)	0 (0.0%)	0 (0.0%)	3 (11.1%)	0.694
Hematuria	0 (0.0%)	0 (0.0%)	0 (0.0%)	0 (0.0%)	>0.999
Proteinuria → nephrotic syndrome	1 (2.4%)	1 (10.0%)	0 (0.0%)	0 (0.0%)	0.357
Nephrotic syndrome → proteinuria	0 (0.0%)	0 (0.0%)	0 (0.0%)	0 (0.0%)	>0.999
Males, n	44	25	3	17	
Proteinuria	1 (2.3%)	0 (0.0%)	0 (0.0%)	1 (5.9%)	0.444
Proteinuria and hypertension	2 (4.5%)	1 (4.0%)	0 (0.0%)	1 (5.9%)	>0.999
Proteinuria and hematuria	5 (11.4%)	4 (16.0%)	0 (0.0%)	1 (5.9%)	0.744
Proteinuria, hematuria, and hypertension	7 (15.9%)	3 (12.0%)	3 (100.0%)	1 (5.9%)	0.003
Nephrotic syndrome	11 (25.0%)	9 (36.0%)	0 (0.0%)	2 (11.8%)	0.161
Proteinuria, nephrotic syndrome, hematuria, and hypertension	3 (6.8%)	0 (0.0%)	0 (0.0%)	3 (17.6%)	0.086
Nephrotic syndrome and hypertension	4 (9.1%)	4 (16.0%)	0 (0.0%)	0 (0.0%)	0.247
Nephrotic syndrome and hematuria	1 (2.3%)	0 (0.0%)	0 (0.0%)	1 (5.9%)	0.444
Nephrotic syndrome, hematuria, and hypertension	4 (9.1%)	0 (0.0%)	0 (0.0%)	4 (23.5%)	0.047
Hematuria	2 (4.5%)	2 (8.0%)	0 (0.0%)	0 (0.0%)	0.571
Proteinuria → nephrotic syndrome	2 (4.5%)	1 (4.0%)	0 (0.0%)	1 (5.9%)	>0.999
Nephrotic syndrome → proteinuria	1 (2.3%)	0 (0.0%)	0 (0.0%)	1 (5.9%)	0.444

The data are presented as the number of individuals n (% of total group/type of inflammation). Inflammation types were compared using Fisher’s exact test; proteinuria → nephrotic syndrome means a progression to nephrotic syndrome from proteinuria; nephrotic syndrome → proteinuria means that the nephrotic syndrome was resolved, but proteinuria remained, which did not meet the criterion for nephrotic syndrome (less than 3.5 g/1.73 m^2^ of body surface area or more than 50 mg/kg). The calculated *p*-value > 0.999 was from Fisher’s exact test. This test was possible in the absence of observations, and the obtained *p*-value could be interpreted as in any other case. Therefore, there was no statistically significant difference in the incidences. Abbreviations: DDD, dense deposit disease; C3GN C3 glomerulonephritis; NC, non-classification criteria.
